# Perifolliculitis capitis abscedens et suffodiens treatment with tumor necrosis factor inhibitors and baricitinib: A case report and literature review

**DOI:** 10.3389/fmed.2023.1132574

**Published:** 2023-03-28

**Authors:** Yuanting Yu, Xiaojie Ding, Fei Guo, Kan Ze, Xiaoying Sun, Xin Li

**Affiliations:** ^1^Department of Dermatology, Yueyang Hospital of Integrated Traditional Chinese and Western Medicine, Shanghai University of Traditional Chinese Medicine, Shanghai, China; ^2^Institute of Dermatology, Shanghai Academy of Traditional Chinese Medicine, Shanghai, China

**Keywords:** PCAS, adalimumab, baricitinib, isotretinoin, case report

## Abstract

**Rationale:**

Perifolliculitis capitis abscedens et suffodiens (PCAS), also known as dissecting cellulitis of the scalp (DCS), is a part of the “follicular occlusion tetrad” that also includes acne conglobate (AC), hidradenitis suppurativa (HS), and pilonidal sinus, which share the same pathogenic mechanism, such as follicular occlusions, follicular ruptures, and follicular infections.

**Patient concerns:**

A 15-year-old boy had multiple rashes on the scalp accompanied by pain.

**Diagnosis:**

The patient was diagnosed with PCAS or DCS based on the clinical manifestations and laboratory examinations.

**Interventions:**

The patient was initially administered adalimumab 40 mg biweekly and oral isotretinoin 30 mg daily for 5 months. Because the initial results were insufficient, the interval between adalimumab injections was extended to 4 weeks, and isotretinoin was changed to baricitinib 4 mg daily for 2 months. When the condition became more stable, adalimumab 40 mg and baricitinib 4 mg were administered every 20 and 3 days, respectively, for two more months until now.

**Outcomes:**

After 9 months of treatment and follow-up, the original skin lesions of the patient were almost cured, and most inflammatory alopecia patches disappeared.

**Conclusion:**

Our literature review did not find any previous reports on treating PCAS with TNF-α inhibitors and baricitinib. Accordingly, we presented the first successful treatment of PCAS with this regimen.

## Introduction

Perifolliculitis capitis abscedens et suffodiens (PCAS) is a rare, refractory dermatitis that tends to recur frequently and primarily affects males with a dark phototype. The lesion, with an extensive infiltrate of neutrophils and lymphoid cells, develops into papules, pustules, and abscesses, which can further advance to the sinus and fistula. The resulting inflammation leads to the formation of a granulation tissue called neutrophilic cicatricial alopecia. PCAS is a member of the “follicular occlusion tetrad,” which also includes acne conglobate (AC), hidradenitis suppurativa (HS), and pilonidal sinus (1). The dominant pathogenic hypothesis involves follicular hyperkeratosis, which induces hair follicle obstruction, forcing the follicle to dilate and rupture, causing secondary infection and subsequent fistulas and abscesses (2). Although the etiology of PCAS is uncertain, several influencing factors, such as neutrophil infiltration, demographic parameter, hormonal risk factors, and the loss of immune tolerance to alloantigens in the hair follicle, may result in inflammation (3).

PCAS treatment is challenging. Isotretinoin and antibiotics are the general protocols for PCAS treatment; however, these drugs are not completely effective in this condition and easily cause relapse after their use is discontinued (3). Surgical excision and X-ray hair removal carry a higher risk of complications. Oral zinc and anti-androgens are utilized in mild to moderate cases (4). Recent case reports and clinical trials have mentioned tumor necrosis factor (TNF) inhibitors as viable PCAS treatments. To explore the potential of the anti-TNF antibody for treating PCAS, we presented the results of a case study and literature review related to an anti-TNF antibody administration to treat PCAS. However, a higher priority may be given to combination therapy because, when considering all of the available therapeutic options, a single treatment frequently has limited efficacy (5). The combined use of Janus kinase (JAK) inhibitors may potentially be a novel treatment scheme for PCAS.

## Case presentation

A 15-year-old Asian boy with a 10-month history of PCAS was referred to our hospital on 17 February 2022. The patient had multiple scalp rashes accompanied by itching, burning, and pain. Prior to hospital admission, the patient received antibiotic treatment with oral minocycline (50 mg twice daily) for 3 months and oral clindamycin (0.15 g four times daily) for 1 month. However, his clinical condition was not well controlled. The patient also consented to an operation to remove the abscess and drain the pus, which left five areas of scar hyperplasia but did not prevent relapses of the inflammatory lesions. The patient denied a history of smoking and family skin appendage disorders. The body mass index (BMI) of the patient was 26.2 (the normal BMI range for men is 18.5–23.9), which is a risk factor for PCAS (6).

**Figure 1:**
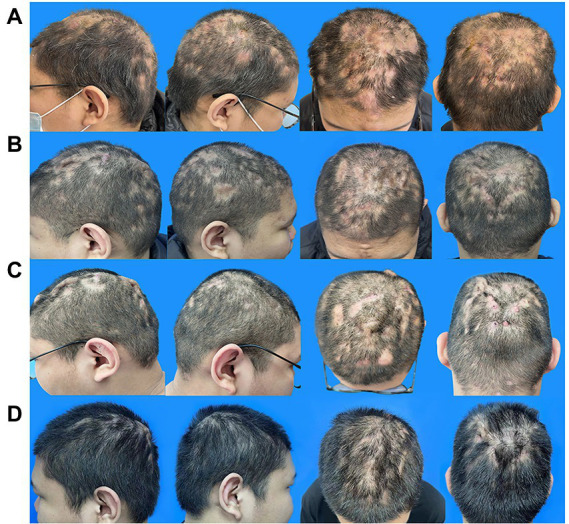
Evolution of the scalp manifestation of the PCAS patient. **(A)** Typical lesions of PCAS on the scalp, nodules, pustules, and alopecia patches prior to adalimumab and isotretinoin therapy on 17 February 2022. **(B)** After 1 month of treatment with adalimumab and isotretinoin on 24 March 2022. **(C)** After 5 months of treatment with adalimumab and isotretinoin on 23 July 2022. **(D)** After changing the treatment regimen to adalimumab and baricitinib for 4 months on November 20, 2022. PCAS, Perifolliculitis capitis abscedens et suffodiens.

Physical examination revealed several fluctuating pustules and prominent erythematous nodules on the scalp, the largest of which was located in the middle of the forehead (approximately 2 cm × 2 cm). The interconnecting sinuses of the patient were filled with malodorous pus, and extensive alopecia patches were located in the parietal-occipital scalp (Figure 1A).

Laboratory tests revealed a leukocyte, neutrophil, and lymphocyte count of 12.7 × 10^9^/L, 9.1 × 10^9^/L, and 19.8%, respectively, and a C-creative protein concentration of 3.38 mg/L. In addition, *Staphylococcus* was isolated from the pus of the lesion, and the drug susceptibility testing showed that Staphylococcus was sensitive to most antibiotics.

PCAS does not have a differential diagnosis or clear diagnostic criteria. Although pathological histological examination allows further observation, the histopathologic characteristics of PCAS usually depend on clinical manifestation. Therefore, the patient was diagnosed with PCAS or DCS according to the tendency, previous diagnosis, treatment history, clinical manifestations, and laboratory examinations of the patient.

Minocycline (50 mg twice daily) was used to control the inflammation from February 17. However, the lesions were not well controlled after 1 week as they were before. Therefore, after excluding contraindications, 80 mg adalimumab on day 0, followed by 40 mg every 2 weeks, and isotretinoin (30 mg daily) were adopted to alleviate the lesions. After 1 month, the patient scalp improved considerably, with fewer fresh pustules and less drainage. Furthermore, the alopecia patches were converted from an extensive wide range to scattered sections and the tenderness markedly subsided (Figure 1B). The laboratory tests revealed leukocyte, neutrophil, and lymphocyte counts of 5.0109/L, 2.5109/L, and 33.8%, respectively, and a C-creative protein concentration of 0.71 mg/L, indicating that the inflammatory response had been controlled.

After a 4-month follow-up, the level of triglycerides increased from 2.29 to 7.47 mmol/L, and the total cholesterol increased from 5.9 to 6.9 mmol/l compared to the values obtained upon patient admission to the hospital on February 18. These changes may be related to isotretinoin and adalimumab administration. In addition, the skin lesions’ improvement seemed to have plateaued (Figure 1C). As a result, fenofibrate (200 mg daily) was administered, and isotretinoin was discontinued to manage the above complications. The frequency of the adalimumab injections was also reduced to every 4 weeks, and baricitinib (4 mg daily) was creatively used as an anti-inflammatory agent and immunity regulator. After 1 month, the triglyceride level and total cholesterol decreased to 4.22 mmol/l and 6.1 mmol/L, respectively.

**Figure 2:**
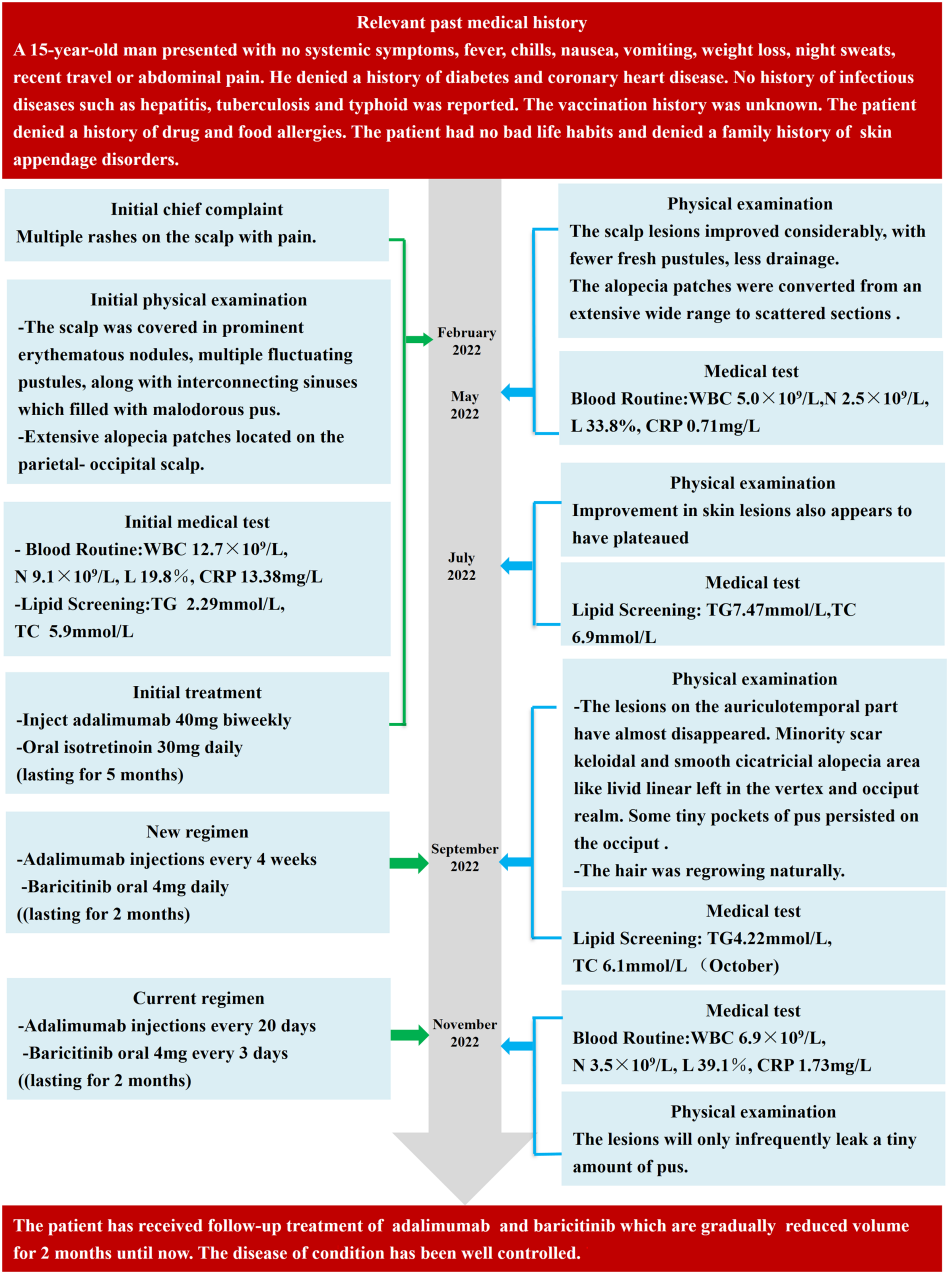
Important dates, periods, and detailed diagnostic process during the PCAS treatment and follow-up periods. WBC, white blood cells; N, neutrophils; L, Lymphocytes; ESR, erythrocyte sedimentation Rate; CRP, C-reactive protein; TG, triglyceride; TC, total cholesterol.

Following the second therapy round, the auriculotemporal lesions almost disappeared, and hair regrowth occurred naturally. However, some tiny pockets of pus persisted on the occiput. After 2 months, the prognosis appeared to have stabilized. Thus, the dosage of baricitinib was reduced to 4 mg every 3 days, and adalimumab injections were administered every 20 days. The lesions painlessly and infrequently leaked a tiny amount of pus after another 2 months of treatment, and most instances of alopecia recovered satisfactorily (Figure 1D). The laboratory tests revealed leukocyte, neutrophil, and lymphocyte counts of 6.9 × 10^9^/L, 3.5 × 10^9^/L, and 39.1%, respectively, and a C-creative protein concentration of 1.73 mg/L. Figure 2 shows the entire course of treatment.

We conducted a literature review in PubMed, Web of Science, Cochrane, and Scopus, valuing the role of TNF inhibitors treating PCAS on 25 February 2023. The database was searched using the terms “Perifolliculitis capitis abscedens et suffodiens” or “dissecting cellulitis” and “tumor necrosis factor” or” baricitinib.” The search yielded 15 citations on 25 February 2023, comprising 19 patients with PCAS treated with TNF inhibitors after ineffective results with conventional treatments. The characteristics of the included studies are summarized in Table 1.

**Table 1:** Clinical characteristics of PCAS patients treated with tumor necrosis factor inhibitors.

No.	Authors	Demographic distribution (Age/Sex/Race)	Duration (Year)	Comorbidity	Previous treatments	TNF-alpha inhibitors	Outcomes (pain/secretions/hair regrowth)
1	Minakawa et al., 2021 (27)	30/M/Asian	12	HS	Oral antibiotics	Subcutaneous adalimumab	-/--/N.D.
2	Spiers et al., 2021 (28)	34/M/N.D.	N.D.	NCA, Ankylosing spondylitis	Oral antibiotics Oral isotretinoin	Subcutaneous 40 mg adalimumab fortnightly Serial scalp excisions	-/--/-
3	Alsantali et al., 2021 (29)	38/M/N.D.	5	NONE	Oral antibiotics Oral isotretinoin	Adalimumab*	-/--/N.D.
4	Kurokawa et al., 2021 (30)	18/M/Asian	6	HS, NCA	Oral antibiotics	Subcutaneous 160 mg adalimumab on day 0, and 80 mg every other week	-/N.D./-
5	Maxon et al., 2020 (31)	37/M/AA	13	Cystic acne	Oral isotretinoin Corticosteroid injections	Subcutaneous 40 mg adalimumab once weekly	-/N.D./-
6	Takahashi et al., 2019 (13)	19/M/Asian	5	HS	Oral antibiotics Zinc supplementation	Adalimumab*	-/--/-
7	Masnec et al., 2018 (32)	26/M/N.D.	N.D.	HS NCA	Oral antibiotics Oral isotretinoin	Subcutaneous 80 mg adalimumab on days 0,1, and 14 followed by 40 mg on day 28 and every week thereafter	-/-/N.D.
8	Mansouri et al., 2016 (33)	48/M/Afro-Caribbean	20	HS	Oral antibiotics Oral isotretinoin Systemic corticosteroids Zinc sulfate Minor surgery	Adalimumab*	-/--/N.D.
9	Mansouri et al., 2016 (33)	27/M/Caucasian	4	NONE	Oral antibiotics Oral isotretinoin Topical and systemic corticosteroids	Infliximab*	-/--/N.D.
10	Martin-García and Rullán, 2015 (34)	30/M/Caucasian	15	NONE	Oral antibiotics. Oral isotretinoin. Minor surgery	Adalimumab*	--/--/-
11	Freja Lærke Sand et al., 2015 (7)	N.D./M/D.M.	N.D.	N.D.	Dapsone Oral isotretinoin Triamcinolone	Subcutaneous adalimumab 40 mg once weekly	Total clearance of the disease
12	Freja Lærke Sand et al., 2015 (7)	N.D./M/D.M.	N.D.	N.D.	Oral antibiotics. Oral isotretinoin Corticosteroid	Subcutaneous adalimumab 40 mg once weekly	Did not respond
13	Lim et al., 2013 (8)	66/M/AA	14	Acne vulgaris. Sycosis barbae	Oral antibiotics. Topical corticosteroids. Rifampin	Subcutaneous adalimumab 40 mg once weekly and oral antibiotics	Total clearance of the disease
14	Navarini et al., 2012 (35)	30/M/N.D.	1	AC, Type2 diabetes mellitus	Oral isotretinoin Corticosteroids. Minor surgery. Rifampicin	Infliximab* (intravenous)	-/--/N.D. Developed a temporary psoriasiform rash after the second intravenous infusion
15	Navarini et al., 2010 (14)	30/M/Caucasian	1	NONE	Oral antibiotics	Adalimumab*	-/-/-. Preexisting pathologic residual structures remained unchanged
16	Navarini et al., 2010 (14)	29/M/Caucasian	4	NONE	Oral antibiotics. Oral isotretinoin	Adalimumab*	-/--/-. Preexisting pathologic residual structures remained unchanged
17	Navarini et al., 2010 (14)	27/M/Caucasian	7	HS	Oral antibiotics. Oral isotretinoin	Adalimumab*	-/N.D./- Preexisting pathologic residual structures remained unchanged. When adalimumab administration was paused, relapse occurred within 4 weeks
18	Brandt et al., 2008 (36)	24/M/N.D.	N.D.	NONE	NA	Infliximab 5 mg·kg^−1^ infused at 8-week intervals	N.D./N.D./-
19	Sukhatme et al., 2008 (37)	39/M/ Caucasian	6	NONE	Minor surgery. Oral isotretinoin	Adalimumab*	--/--/-

N.D.: not described; AA: African-American; NCA: nodulocystic acne; AC: acne conglobata; HS: hidradenitis suppurativa; Adalimumab*: 80 mg Adalimumab was administered on day 0, followed by 40 mg weekly (treated according to the recommended psoriasis regimen); Infliximab*: 5 mg kg^−1^ infliximab was administered in weeks 0, 2, and 6, followed by 8-week intervals (treated according to the recommended psoriasis regimen); -: relieve, --: Cease.

In the selected studies analyzed, all patients were males, with an average age of 32.47 ± 11.35 years (mean ± SD). The average duration of PCAS history was 8.1 ± 5.7 years (mean ± SD). The co-morbidity between PCAS and HS was 31.6%. Isotretinoin combined with antibiotics was the first-line treatment in 47.4% of PCAS cases. However, no obvious improvement was observed. Among the patients with PCAS administered TNF inhibitors, only one experienced no response, and two reported total clearance of the disease (7, 8). The remaining cases had marked pain reduction and inflammation relief. In particular, 89.5% of the patients with PCAS had pain relief, and 63.2% demonstrated complete cessation of pus. In addition, hair regrowth occurred in 63.2% of patients treated with TNF inhibitors.

On 25 February 2023, we also retrieved the English-language literature by searching the WHO trials register and Clinicaltrials.gov (clinical trial registries). Our search strategy focused on combination therapies providing a TNF inhibitor (adalimumab, infliximab, certolizumab pegol, etanercept, or golimumab) with a JAK inhibitor (baricitinib, tofacitinib, ruxolitinib, upadacitinib, or filgotinib). We found a single randomized controlled trial currently recruiting participants to study baricitinib in combination with adalimumab to treat RA (NCT04870203).

## Discussion

PCAS is a well-known chronic and disfiguring disease. Current treatment for PCAS is non-standardized. In this case report, conventional integrated treatments with antibiotics, isotretinoin, and minor surgery failed to induce complete remission. The initial treatment regimen with isotretinoin and adalimumab led to the remnant of sinuses and areas of alopecia with hyperpigmentation, accompanied by elevated blood lipids. Eventually, we attempted an unprecedented approach and jointly administered adalimumab and baricitinib, which have never been explored as a combination regimen for treating PCAS.

A systematic literature review demonstrated that isotretinoin was administered to 53% of patients with PCAS, and a significant response was observed in half of those PCAS cases. However, the relapse rate was 19%. Antibiotics were administered to limit the disease in patients with milder PCAS conditions (4). Isotretinoin is assumed to normalize the skin and follicular apparatus to reduce the abnormal immune response. Antibiotics are effective supplements for moderating the inflammatory process, particularly during suspected secondary bacterial infection, like in our case. However, the unknown etiology, such as the inflammatory pathways in the skin of patients with PCAS, is responsible for the unsatisfactory recovery rate (9).

TNF-α is a cytokine involved in the pathogenesis of some inflammatory and autoimmune diseases. Therapeutic drugs act as antagonists by blocking the interaction of TNF-α with the type 1 (TNFR1) and type 2 (TNFR2) receptors. TNF-α inhibitors, including adalimumab, infliximab, golimumab, etanercept, and certolizumab, have been approved for clinical use (10). Among them, adalimumab and infliximab are frequently administered for treating PCAS. Adalimumab is a fully human monoclonal antibody, whereas infliximab is a recombinant chimeric monoclonal antibody (11). A retrospective evaluation revealed that the off-label use of TNF-α inhibitors to treat “follicular occlusion tetrads,” which share the same pathogenesis with PCAS, had an extent impact on the outcome (7). Furthermore, adalimumab is a first-line biologic for patients with moderate-to-severe HS. TNF-α is the main driver of the inflammatory pathways predominant in HS skin lesions (12). From a clinical viewpoint, TNF-α inhibitors dramatically affected skin lesions and inflammatory symptoms in individual PCAS cases (13). Accordingly, adalimumab is speculated to be beneficial for treating PCAS. TNF-α inhibitors are novel biologics therapies and have garnered remarkable attention in recent years. However, these inhibitors have some limitations in scar restoration and hair regrowth (13). TNF-α inhibitors can minimize secretion, reduce inflammation, and relieve pain. Notably, the structural alteration of the tissue or promotion of blanket recovery by the TNF-α inhibitors is uncommon (14). Herein, we co-administered adalimumab and baricitinib to achieve a better outcome in a case of PCAS.

The JAK signal transducers and activators of transcription (STAT) are essential signaling pathways in various inflammatory diseases (15). JAK–STAT inhibition inhibits cytokine signaling to reduce the serum C-reactive protein levels. In mammals, there are four types of JAK family proteins: JAK1, JAK2, JAK3, and tyrosine kinase 2 (TYK2). Baricitinib is an oral selective and reversible inhibitor of JAK1 and JAK2 that inhibits JAK enzyme response to cytokine and the growth factor receptor stimulation to influence downstream hematopoiesis and immune cell function (16). Based on mounting clinical trials, JAK inhibitors have a promising prospect in dermatology and are effective for treating alopecia areata, atopic dermatitis, and psoriasis, as their relevant cytokines rely on the JAK–STAT pathway (17–20). In 2022, the safety and efficacy findings of two phase II studies demonstrated the positive role of the JAK1 inhibitor INCB054707 in HS patients (21). Therefore, JAK1 may be a potential drug target in PCAS. In June 2022, the US Food and Drug Administration (FDA) approved baricitinib oral tablets to treat adult patients with severe alopecia areata, a disorder characterized by inflammatory, nonscarring patchy hair loss. As both alopecia areata and PCAS are types of inflammatory alopecia, baricitinib may have a similar therapeutic effect on PCAS. Baricitinib reduces inflammation in HS, and alopecia areata, and it displays potential as a drug for treating PCAS by dampening the inflammatory response. In our patient with PCAS, a significant reduction in scarring and visible hair regrowth at the site of the lesion were observed after the administration of baricitinib.

TNF-α and JAK inhibitors are used as biologics for treating psoriasis, inflammatory bowel disease, RA, and other diseases by inhibiting inflammatory signaling pathways. In some cases, the cascade response between the two inhibitors has been validated. Kandhaya-Pillai et al. demonstrated that JAK inhibitor interrupted the interactive loop between the synergy of TNF-α and IFN-γ to prevent hyper-inflammation and normalize SARS-CoV-2 entry receptor expression (22). In addition, a study showed that TNF-α was significantly reduced in adult patients with systemic lupus erythematosus treated with baricitinib (23). By pathway analysis, Krisztina et al. have revealed the involvement of TNF and JAK–STAT signaling in the complex regional pain syndrome, which presents severe chronic pain, hypersensitivity, and inflammation. Baricitinib can also compensate for inadequate response to tumor necrosis factor inhibitors (TNFis). In a retrospective therapeutic drug monitoring case series, patients with HS, with anti-adalimumab antibody, had significantly lower serum adalimumab levels. In addition, the patients with HS in the supratherapeutic groups were treated for a significantly longer time period than the therapeutic patients (24). Baricitinib has also been reported to outperform TNFis in the treatment of RA (25).

In summary, we administered baricitinib to patients with PCAS to interrupt TNF-α and IFN-γ inflammatory pathways and help them compensate for their suboptimal response to adalimumab treatment. Our results showed that this combinatorial treatment was indeed beneficial. Given the potential for increased frequency of serious adverse events (including heart disease, blood clots, infections, and anemia) when combining targeted therapies, we are very cautious in setting the dosage of the two medications. According to a meta-analysis published in 2022, JAK inhibitor was discontinued more often among patients with RA for adverse events, and less often for inefficacy compared to TNF inhibitor (26). Treatment continues to be dominated by adalimumab, with baricitinib playing an adjuvant role. Although combining the two drugs increases the risk of an adverse event, it remains worth trying within a manageable range for refractory conditions in which patients have experienced multiple treatment failures. Baricitinib alone may also be a viable option, but we are testing it under the premise that adalimumab is effective against HS, and adalimumab did provide relief in this case. We thus opted to continue with combination therapy for our second round of treatment.

A combination of treatment modalities is often necessary to optimize treatment outcomes. We provide a feasible solution for patients who do not fully respond to TNF-α inhibitors, or who have tenacious scarring and hair loss sequelae. However, the role of TNF-α and JAKs in the pathogenesis of PCAS has not been established. Our experience of treating PCAS is based on the HS and AA populations. Considering the relative lack of studies on the immunology of this condition, a fundamental understanding of immunological dysregulation in PCAS is urgently required. In the future, more standard evaluation criteria, and robust studies, including randomized control trials, are required to determine the preferred treatment options.

## Author’s note

The authors have read the CARE Checklist (2016), and the manuscript was prepared and revised according to the CARE Checklist (2016).

## Data availability statement

The original contributions presented in the study are included in the article/supplementary material, further inquiries can be directed to the corresponding author/s.

## Ethics statement

Ethical review and approval was not required for the study on human participants in accordance with the local legislation and institutional requirements. Written informed consent to participate in this study was provided by the participants’ legal guardian/next of kin. Written informed consent was obtained from the minor(s)’ legal guardian/next of kin for the publication of any potentially identifiable images or data included in this article.

## Author contributions

YY conducted the literature search and drafted the manuscript. XD was responsible for the clinical treatment of the patient, meanwhile she revised and polished the thesis in post. FG were involved in patient management and contributed to metadata acquisition and interpretation. KZ, XS, and XL critically reviewed and revised the manuscript. All authors contributed to the article and approved the submitted version.

## Funding

This study was funded by The National Natural Science Foundation of China (No. 82074427), the Clinical Research Plan of SHDC (No. SHDC2020CR3097B), the Shanghai Sailing Program (No. 20YF1450400), Shanghai Three-Year Action Plan to Further Accelerate the Inheritance and Innovative Development of Chinese Medicine (2021–2023) [No. ZY(2021–2023)-0302], and the Three-Year Action Plan for Further Accelerating the Inheritance and Innovative Development of Chinese Medicine in Shanghai (2021–2023) [No. ZY(2021–2023)-0209-13].

## Conflict of interest

The authors declare that the research was conducted in the absence of any commercial or financial relationships that could be construed as a potential conflict of interest.

## Publisher’s note

All claims expressed in this article are solely those of the authors and do not necessarily represent those of their affiliated organizations, or those of the publisher, the editors and the reviewers. Any product that may be evaluated in this article, or claim that may be made by its manufacturer, is not guaranteed or endorsed by the publisher.

## Abbreviations

PCAS: Perifolliculitis capitis abscedens et suffodiens
DCS: Dissecting cellulitis of the scalp
AC: Acne conglobate
HS: Hidradenitis suppurativa
TNF: Tumor necrosis factor
TNFR1: Tumor necrosis factor receptor 1
TNFR2: Tumor necrosis factor receptor 2
JAK: Janus kinase
STAT: Signal transducers and activators of transcription
TYK2: Tyrosine kinase 2
FDA: US Food and Drug Administration
